# Study of CFRP Laminate Gradually Modified throughout the Thickness Using Thin Ply under Transvers Tensile Loading

**DOI:** 10.3390/ma17102388

**Published:** 2024-05-16

**Authors:** Hossein Malekinejad, Farin Ramezani, Ricardo J. C. Carbas, Eduardo A. S. Marques, Lucas F. M. da Silva

**Affiliations:** 1 Instituto de Ciência e Inovação em Engenharia Mecânica e Engenharia Industrial (INEGI), Rua Dr. Roberto Frias, 4200-465 Porto, Portugal; hmalekinejad@inegi.up.pt (H.M.); farinramezani@gmail.com (F.R.); 2 Departamento de Engenharia Mecânica, Faculdade de Engenharia (FEUP), Universidade Do Porto, Rua Dr. Roberto Frias, 4200-465 Porto, Portugal; emarques@fe.up.pt (E.A.S.M.); lucas@fe.up.pt (L.F.M.d.S.)

**Keywords:** composite materials, thin plies, transverse tensile, fracture, RVE

## Abstract

The use of thin-ply composite materials has rapidly increased due to their tailorable mechanical properties and design flexibility. Considering an adhesively bonded composite joint, peel stress stands out as a key contributor leading to failure among other primary stress factors. Therefore, the reinforcement of carbon fiber-reinforced polymer (CFRP) laminates throughout the thickness could be considered as an approach to improve the joint strength. Using thin plies locally between the conventional CFRP layers in a laminate can enhance the strength, as the sudden change in stiffness means that the load transfer is not monotonous. Consequently, the following study examined the effect of altering thin plies gradually throughout the thickness on the behaviour of the CFRP laminates when subjected to transverse tensile loading. To achieve this goal, the CFRP laminates were gradually modified by using different commercially accessible prepreg thin plies, leading to an improved overall structural performance by reducing stress concentrations. Besides conducting an experimental study, a numerical assessment was also carried out utilizing Abaqus software with a Representative Volume Element (RVE) at the micro scale. The comparison of reference configurations, which involved various thin plies with different thicknesses and traditional CFRP laminates, with the suggested gradual configuration, demonstrated a notable enhancement in both strength and material cost. Furthermore, the proposed RVE model showed promising capability in accurately forecasting the strength of fabricated laminates.

## 1. Introduction

Composite materials have gained widespread acceptance in the automotive, aerospace, and marine sectors owing to their distinct attributes such as an elevated strength-to-weight ratio, effective heat insulation, remarkable corrosion resistance, and efficient sound absorption [1]. These materials typically consist of two primary components, fibers and a matrix [2]. In composite materials, the fibers contribute to the material’s stiffness and strength, while the second component, often known as resin or “matrix,” provides cohesion to the fibers, enabling the composite to withstand shear loads [3,4]. The combination of these components makes a new material with completely different chemical and physical properties compared to the raw materials [5]. A transverse tensile load applied to composite materials is primarily carried by the least robust component of the composite (matrix), ultimately leading to delamination. This commonly occurs in composite joints that are bonded with adhesive when peel stress is applied to the composite adherends [6,7]. Delamination failure under transverse tensile loads has the potential to degrade both the mechanical properties and stiffness of the structure, ultimately contributing to catastrophic failure [8,9,10].

Numerous approaches have been utilized to enhance the transverse tensile strength of composite materials or increase their resistance to delamination. A significant advancement in the modification of composites field relates to the emergence of thin ply laminates. Thin plies are typically characterized as composite materials with ply thicknesses that are less than 100 μm and a ply areal weight below 100 g/m^2^ [11]. Thin plies have attracted great attention due to their ability to enhance design flexibility and provide improved mechanical performance when subjected to different types of loads including static, impact, and fatigue loading. Furthermore, the utilization of thin plies leads to a reduction in shear stress, primarily attributed to the increased number of layers and associated interfaces [12]. Having discussed another advantage earlier, it is worth noting that thin plies have also been fabricated through a specific resin spreading process [13]. This process ensures a more uniform distribution of fibers within the resin, resulting in a decrease in resin-rich areas [14]. Hence, the likelihood of matrix cracking in thin plies is significantly lower compared to conventional composites where fibers are not as uniformly distributed as in thin plies. Furthermore, numerous research studies underscore additional benefits of thin plies, such as their improved durability and electrical resistance characteristics, which make them particularly intriguing for further investigation [15,16]. 

Several attempts have been made in various studies to enhance the performance of composite laminates by employing thin plies as reinforcements. However, these effort primarily focus on reinforcing local areas and applying thin plies at different place throughout the thickness of composite laminate separately. Although improvements in out-of-plane strength have been demonstrated by locally reinforcing the laminate using thin plies [6], the non-monotonous load transfer due to sudden changes in material toughness and stiffness throughout the thickness can be a barrier to achieving the maximum benefit of thin plies.

The study of toughened laminate gradually modified throughout the thickness is a research area that focuses on the analysis and optimization of composite laminates with varying material properties throughout the thickness. The aim is to reduce the stress concentration caused by uniform load transfer throughout the thickness, consequently improving the mechanical performance of composite materials. 

This research delves into the innovative idea of creating a graded CFRP laminate by incorporating varying thicknesses of thin plies throughout the laminate’s thickness. To achieve this goal, various commercially available thin ply and conventional composite prepregs are integrated into the laminates. This includes thin thin plies and intermediate thin plies with thicknesses of 0.015 mm and 0.070 mm, respectively, along with conventional composites with a thickness of 0.150 mm. The study examined and compared the performance of proposed graded FRP laminates under transverse tensile stress with standard benchmark configurations. 

Simulating the behaviour of FRP laminates under transverse tensile loading poses a persistent challenge due to the occurrence of stress concentration, which is difficult to accurately account for in numerical simulations. In this research, changes in fiber distribution resulting from the utilization of different materials (conventional and thin ply) make the simulation even more complicated. Isolating the effect of fiber distribution on the composite performance may not be possible with commonly used numerical models. Therefore, computational micromechanics has recently emerged as a promising tool for predicting how fiber distribution and also shape affect the strength of composite laminates under different loading conditions [17,18]. This approach relies on the numerical simulation of the mechanical response of a representative volume element (RVE) within the composite microstructure. Consequently, in this study, an RVE model is developed to simulate the transverse tensile strength of all experimentally tested configurations. To depict the strength and failure of composite laminates numerically, the concrete damage plasticity (CDP) model in Abaqus software 2017 was utilized to simulate the plastic behaviour of the matrix as a quasi-brittle material. CDP is commonly utilized for modelling epoxy matrices as quasi-brittle materials, accounting for both the pressure-sensitive flow stress of epoxy under compression and its tendency to behave in a brittle manner when subjected to tension [19,20]. Additionally, cohesive failure criteria were employed to capture the separation of the fiber/matrix interface, alongside the use of RVE.

## 2. Experimental Study

### 2.1. Materials

All materials utilized in the subsequent study were chosen to accurately represent those commonly employed in the aerospace sector [21]. A unidirectional carbon-epoxy prepreg, commercially known as Texipreg HS 160T700 (Seal Spa in Legnano, Italy), with a ply thickness of 0.15 mm was used as the conventional composite. Unidirectional composite prepregs with the commercial reference of NTPT-TP415 fabricated by North Thin Ply Technology of Renens, Switzerland were used as the thin ply material. Two distinct thin ply prepregs with ply thicknesses of 0.015 mm and 0.07 mm were employed to perform as thin thin ply and intermediate thin ply, respectively [14]. 

### 2.2. Configurations

**Figure 1:**
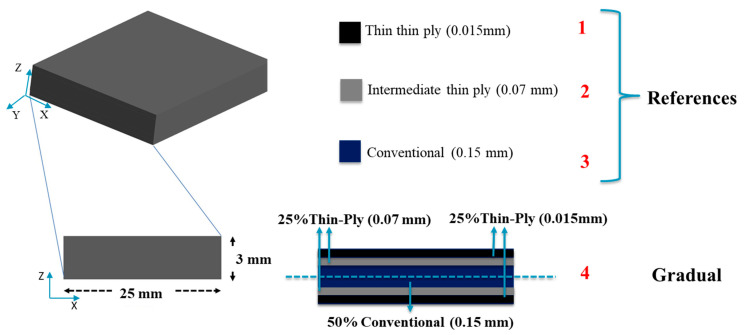
Schematic design of the reference and gradual composites laminates.

Initially, reference composite laminate adopting each of the three materials mentioned in Section 2.1 were manufactured as benchmark configurations. Subsequently, gradual laminates were created with a combination of conventional, thin, and intermediate thin-ply composites, with the following distribution: 50% conventional composite in the middle and 25% of each thin and intermediate thin plies in the outer layers seeking symmetry of the final laminate, as illustrated in Figure 1.

### 2.3. Manufacturing Process 

**Figure 2:**
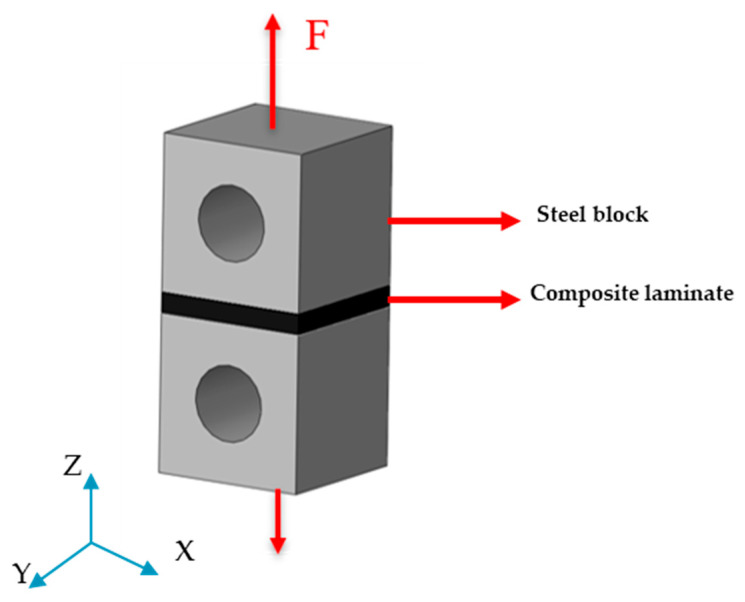
Schematic design of steel blocks attached to conventional composite laminates and loading conditions.

The hand lay-up method was employed for all of the configurations illustrated in Figure 1. The stacking of the plies layer by layer persisted until the desired laminate thickness was attained. As shown in Figure 1, for the gradual laminate, 50% of the overall thickness of the composite laminate consists of the conventional composite, and the remaining is dedicated to thin and intermediate thin ply in equal percentages. To ensure that all the manufactured laminates maintain a consistent thickness of 3 mm, an aluminum mold was utilized. A release agent was applied to the mold to facilitate the demolding process. Subsequently, the plates were subjected to curing in a hot press at the following conditions advised by the manufacturer: 30 bar pressure and 130 °C temperature for a duration of 2 h. Following the curing process, the plates were trimmed to achieve the specified dimensions of 25 × 25 mm^2^, and lastly, the manufactured laminates were attached to steel blocks as a part of the transverse tensile test set-up (Figure 2). The process of attaching laminates to steel blocks was carried out using AV138 epoxy resin along with HV 998 as a hardener manufactured by Huntsman Cambridge (UK). As per the manufacturer’s technical datasheet for AV138, the curing procedure required keeping the assembly at room temperature for a duration of 24 h.

### 2.4. Surface Treatment

**Figure 3:**
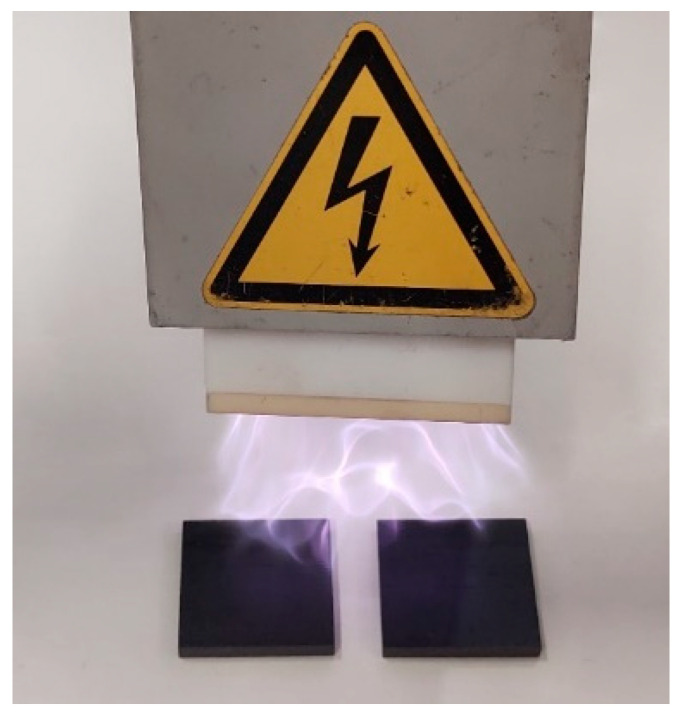
Surface treatment of composite laminate using plasma.

As discussed in Section 2.3, the composite laminates were bonded to steel blocks. For achieving optimal adhesion of specimens, it is essential to employ distinct surface preparation methods, including one for the composite laminate and another for the steel surfaces. The steel surface is first subjected to sanding using a sanding machine, followed by thorough cleaning with acetone to eliminate any contaminants. Regarding the composite laminate, initially, the composite laminate’s surfaces underwent preparation involving light sanding and subsequent cleansing with acetone to eliminate any contaminants. Following this, a plasma treatment (see Figure 3) was conducted on the composite surfaces to enhance their surface energy before the bonding process [22]. 

### 2.5. Scanning Optical Microscope

**Figure 4:**
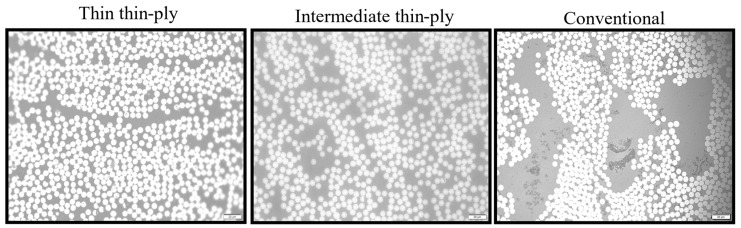
Distribution of fibers that corresponded to different composites observed under a microscope.

In accordance with existing literature and prior studies on diverse composite materials, it is noted that the bonding at the interface between plies significantly influences the overall composite laminate properties [23,24]. Consequently, all mentioned materials were examined by employing an optical microscope to observe their cross-sections. To capture high-quality photos, surface preparation is essential before observation. Each sample underwent a process where they were embedded in epoxy resin powder, cured using an embedding machine, and subsequently polished on an automated polishing machine utilizing wet sandpaper with incrementally increasing grit sizes (P400, P800, and P1200). The laminates were then observed under an optical microscope with different zoom scales. No highly visible macro cracks or voids were observed and the quality of samples was acceptable. Figure 4 shows the distribution of fiber observed under the microscope. Figure 4 proves the efficiency of the preparation process and quality of the manufactured laminates that are produced without voids and high porosity. 

### 2.6. Testing Condition

**Figure 5:**
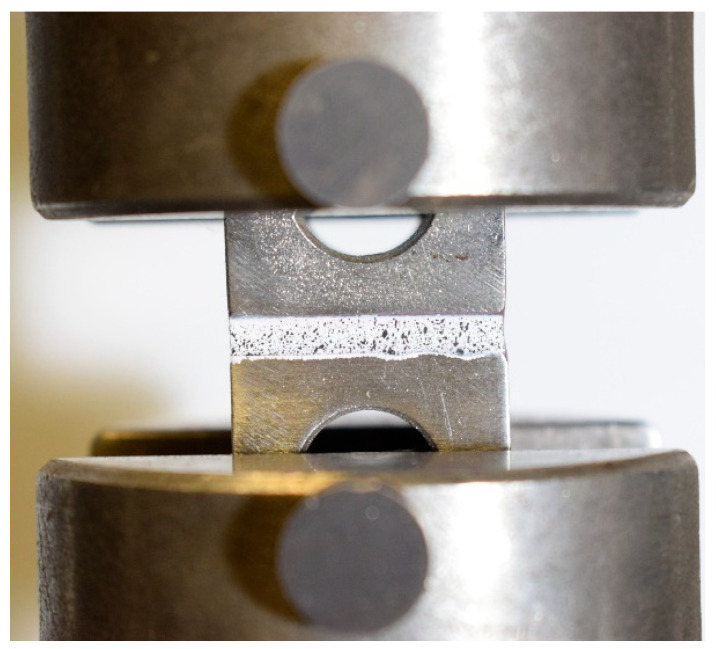
Specimens under transverse tensile test.

A transverse tensile test was performed on all specimens using the Instron 3367 machine sourced from Norwood, MA (USA), with a testing speed set at 1 mm/min (quasi static). Each configuration was subjected to a minimum of five repetitions. All tests were performed under laboratory ambient conditions (room temperature of 24 °C, relative humidity of 55%). Figure 5 illustrates a specimen under loading and the test set-up.

## 3. Numerical Study

### 3.1. Generating Representative Volume Element 

**Figure 6:**
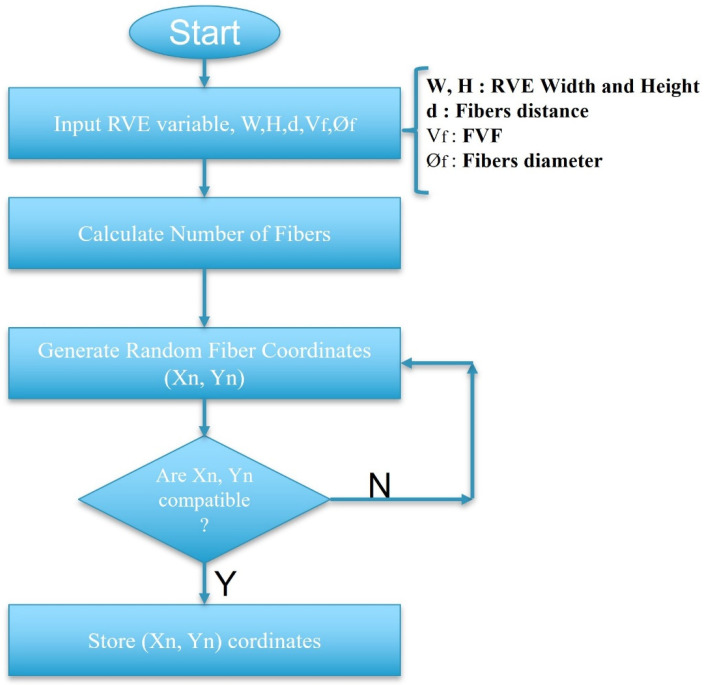
The flowchart of RVE generation.

**Figure 7:**
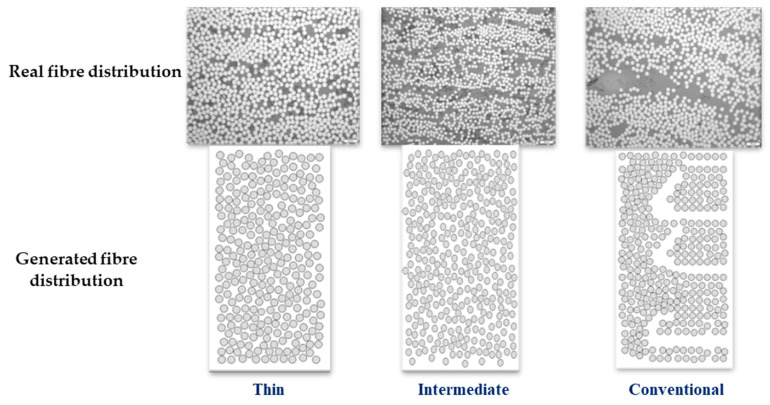
Generated single RVEs.

The RVE model was developed using the ABAQUS software to investigate the behaviour of the studied configuration at the micro-scale. In the beginning, a small-scale RVE measuring 0.21 × 0.15 mm^2^ was created. Following that, the primary RVEs were modelled by combining the smaller ones to achieve the desired dimensions of 1.5 × 1.5 mm^2^. To achieve the most accurate distribution of fibers, a Python-based algorithm (python 3.11) was created. As shown in Figure 6, the algorithm takes dimensions of the RVE, fiber diameter (see Table 1), and fiber volume fraction, as inputs and generates a randomly distributed RVE with the desired fiber configuration with a defined fiber distance (reaching high fiber volume fractions (up to 65%)). The primary aim of the generated algorithm was to ensure that it closely replicated the distribution patterns observed in both the thin plies and the conventional composite material (see Figure 7). As mentioned in Section 2.5, electro optical microscopy analysis was conducted to examine the distribution of resin and fibers in thin plies and conventional composites and also to obtain the fiber volume fraction and fiber diameters, which serves as input data for the fiber distribution algorithm. The noticeable spaces between fibers depicted in Figure 7 for conventional composites were mainly a result of the random fiber distribution algorithm, rather than intentional design. In conventional composites, the spacing between adjacent fibers was deliberately set larger compared to the distance in thin plies. While the algorithm was not able to perfectly replicate conventional distributions, minor modifications were made to position the fibers as realistically as possible, resembling actual distributions found in conventional composites.

**Figure 8:**
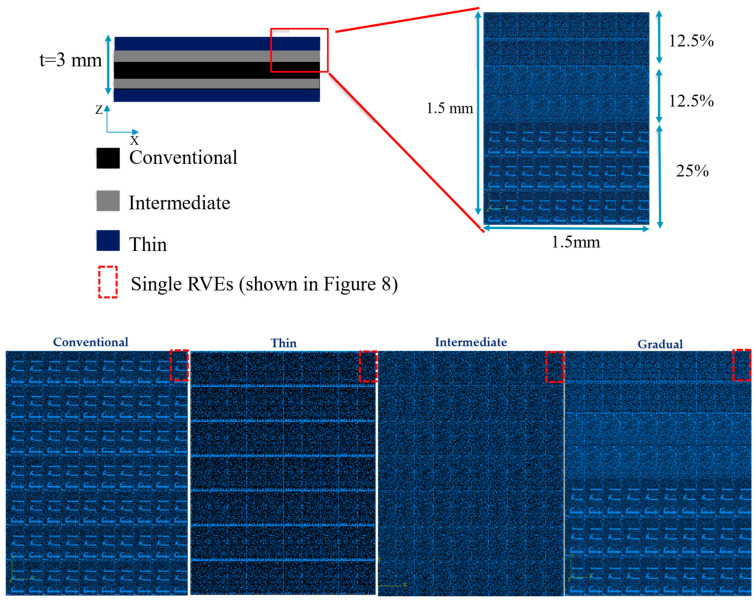
The representative model of reference and gradual configuration.

The next stage involved the simulation of four models by simply reproducing the main RVE for each representative configuration. Among these, three were designed as benchmarks, each representing the distribution characteristics of two distinct types of thin plies and a conventional composite material. The fourth model was created to demonstrate the gradual laminate configuration. As shown in Figure 8, each RVE corresponding to each configuration was constructed by assembling the necessary quantities of single RVEs to achieve the desired dimensions. 

**Figure 9:**
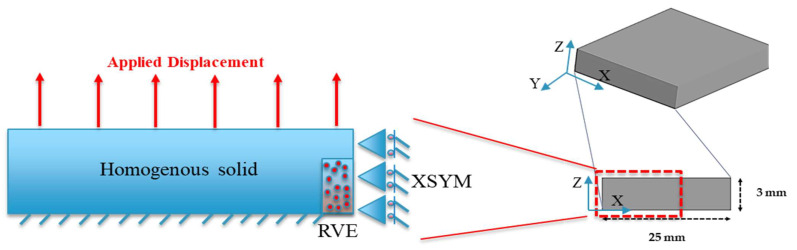
Boundary conditions applicable in RVE models.

When it comes to setting boundary conditions, there are three options available. The first approach involves embedding the conditions directly [25]. The second approach involves simulating a quarter of the entire model while applying symmetry boundary conditions [6], and the last one involves using the periodic boundary condition [26]. As illustrated in Figure 9, the embedded boundary condition was employed due to the simplicity of the conduction process in this study and decrease in the calculation cost. For displacement loading control simulation, a 2D model as well as a static general step were employed. The model was meshed using two-dimensional, four-node bilinear plane stress quadrilateral elements. It is important to note that generating a mesh for such complex geometry poses challenges, often resulting in errors in Abaqus. While the number of elements varies slightly for each configuration, a global mesh size of approximately 0.005 mm was utilized for both matrix and fibers. The elastic properties of the fibers and matrix components of each RVE are presented in Table 1.

**Table 1:** Mechanical and physical properties of conventional and thin ply composites [6,27,28].

Materials	E Resin (GPa)	E Fiber (GPa)	Fiber Diameter (μm)
Conventional	5.07	230	7
Thin	5.07	294	5
Intermediate	5.07	294	7

### 3.2. Matrix Damage Plasticity Criteria Model

**Figure 10:**
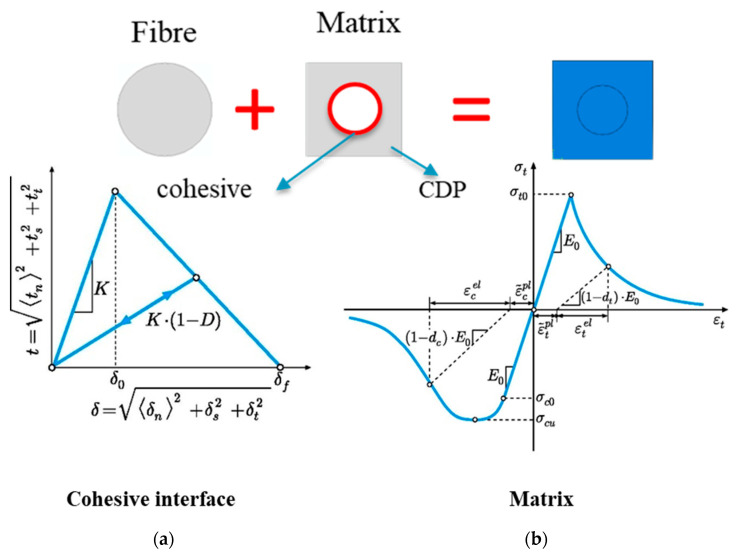
Implementation of (**a**) the cohesive interface model and (**b**) concrete damage plasticity (CDP).

To assess the strength characteristics of each configuration, a combination of concrete damage plasticity (CDP) and cohesive failure criteria were applied [29]. Specifically, to predict potential failures within the matrix, the model employed concrete damage plasticity (CDP). The plastic-damage model utilized in ABAQUS is based on the formulations introduced by Lubliner et al. [19] and Lee and Fenves [20]. These models prove to be applicable for the examination of materials exhibiting quasi-brittle characteristics. As per this constitutive model, the material demonstrates brittle behaviour under tensile stress, while it behaves in an elastoplastic manner under compressive loads. Consequently, the material’s tensile behaviour follows a linear elastic pattern until their reaches the tensile failure stress (*σ_t_*_0_), characterized by the elastic modulus (*E_m_*) and Poisson’s ratio (*ν_m_*). Beyond this point, a quasi-brittle softening phenomenon is evident, governed by the fracture energy, *G_c_*. Conversely, when subjected to compression loading, the behaviour remains linear until reaching the initial yield limit, *σ_c_*. Subsequently, a strain hardening phase ensues until the ultimate stress value (*σ_cu_*) is reached, as illustrated in Figure 10. The parameters for the matrix plasticity/damage model employed in the simulations are provide in the Table 2. When unloading from any point on the strain softening branch, the matrix experiences reduced elastic stiffness (degradation), characterized by damage variables $d_{t}$ and $d_{c}$, as shown in Equations (1) and (2), which are assumed to be influenced by plastic strains.

(1) $$d_{t}=d_{t}\left(\varepsilon_{t}^{pl}\right);\;\;0\leq d_{t}\leq 1$$

(2) $$d_{c}=d_{c}\left(\varepsilon_{c}^{pl}\right);\;\;0\leq d_{t}\leq 1$$

In a generalized form, a single degradation damage variable *D* can be employed to depict both tensile and compressive degradation responses, provided it is defined in Equation (3) [20].

(3) $$D=1-(1-D_{t})(1-D_{c});\;\;0\leq D\leq 1$$

The damage variables range from zero, indicating undamaged material, to one, indicating complete loss of strength. If $E_{0}$ represents the initial (undamaged) elastic stiffness of the material, the stress–strain relationships under uniaxial tension and compression loading are depicted in Equations (4) and (5) [30].

(4) $$\sigma_{t}=\left(1-d_{t}\right)E_{0}(\varepsilon_{t}-\varepsilon_{t}^{p})$$

(5) $$\sigma_{c}=\left(1-d_{c}\right)E_{0}(\varepsilon_{c}-\varepsilon_{c}^{p})$$

**Table 2:** Matrix damage plasticity model input data [28,31,32].

*E_m_* (GPa)	*ν_m_*	*σ_t_*_0_ (MPa)	*σ_c_*_0_ (MPa)	*σ_cu_* (Pa)	*G_c_* (J/m^2^)
5.07	0.35	121	176	180	90

### 3.3. Fiber–Matrix Interface Model

Additionally, cohesive elements were introduced to simulate the phenomenon of fiber/matrix debonding (see Figure 10). The mixed-mode cohesive traction separation (Equation (6)) is defined by the following stress criterion:

(6) $${\left\{\frac{\left\langle t_{n}\right\rangle }{t_{n}^{0}}\right\}}^{2}+{\left\{\frac{t_{s}}{t_{s}^{o}}\right\}}^{2}+{\left\{\frac{t_{t}}{t_{t}^{o}}\right\}}^{2}=1.0$$

In the above equation, *t_n_* is normal traction and *t_s_*, *t_t_* are the shear components of the traction vector. *N*, *S* and *T* are normal and shear strengths, respectively, while the shear component is assumed to be equal [31,33]. The damage progress is defined using the Benzeggagh–Kenane [34] relation as shown in Equation (7) below:

(7) $$G_{C}=G_{IC}+(G_{IIc}-G_{IC})({\frac{G_{II}}{G})}^{\eta_{BK}}$$

where the $\eta_{BK}$ is the Benzeggagh–Kenane power exponent; *G_C_* represent the critical shear and normal fracture energy [34]. Further details and relevant parameters can be found in Table 3.

## 4. Results and Discussion

### 4.1. Experimental Results and Discussion

#### 4.1.1. Fiber Volume Fraction (FVF)

Attaining the precise fiber volume fraction contributes to increasing the overall validity of the numerical model. To determine the fiber volume fraction for each laminate, scanning optical microscopy was employed, as described in Section 2.5. Microscopic images were captured and then subjected to analysis through both manual examination and the utilization of commercially available image processing software (MIP.4 Full) [35]. For manual calculation, Equation (8) is used, where n represents the number of fibers, *A_f_* corresponds to the fiber section area and *A_t_* represents the total area.

(8) $$\mathrm{FVF}=\frac{n{\;\times \;A}_{f}}{A_{t}}$$

**Figure 11:**
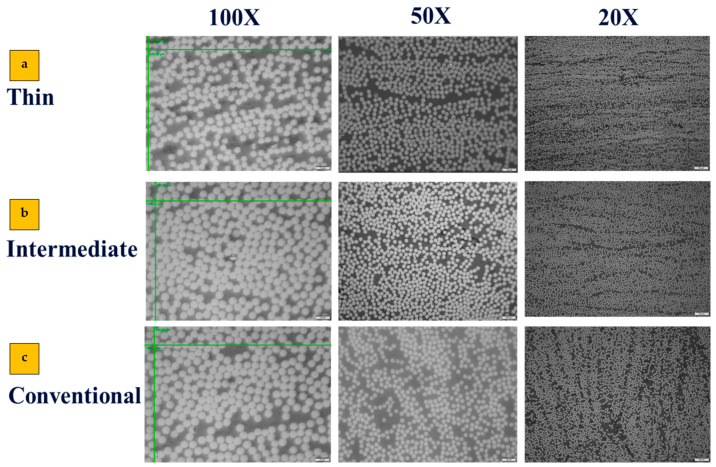
Microscopic images at different scales of (**a**) thin, (**b**) intermediate; (**c**) conventional composites.

**Figure 12:**
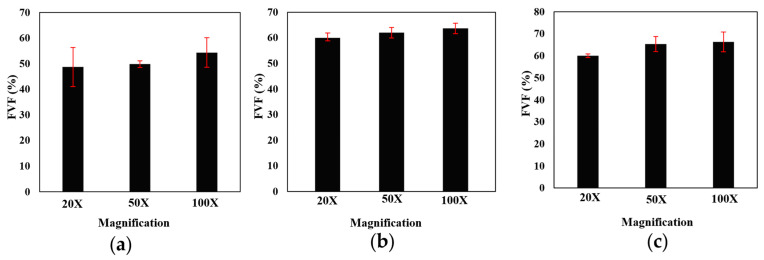
FVF calculated using image processing of (**a**) thin, (**b**) intermediate; (**c**) conventional composite.

**Figure 13:**
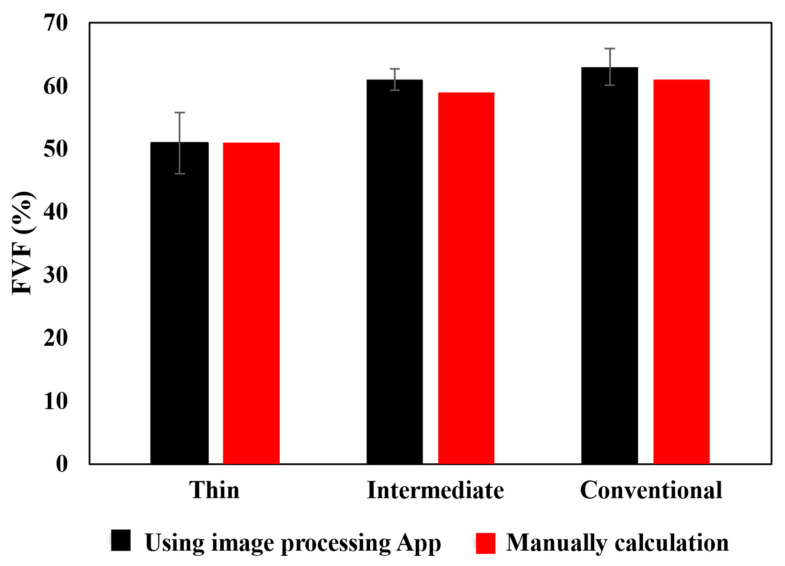
Comparison between image processing and manual calculation of FVF.

This dual approach was undertaken to ensure the accuracy and reasonableness of the calculated fiber volume fractions. In the case of each laminate, images were captured at three distinct levels of zoom, 20×, 50×, and 100× (see Figure 11). Additionally, for each zoom level, three separate images were taken in three different regions. As depicted in Figure 12, the fiber volume fractions obtained across a range of scales from 20× to 100× are roughly consistent. Figure 13 displays the average outcomes for all magnifications considered, acquired from both manual techniques and image processing software.

#### 4.1.2. Transverse Tensile Tests

**Figure 14:**
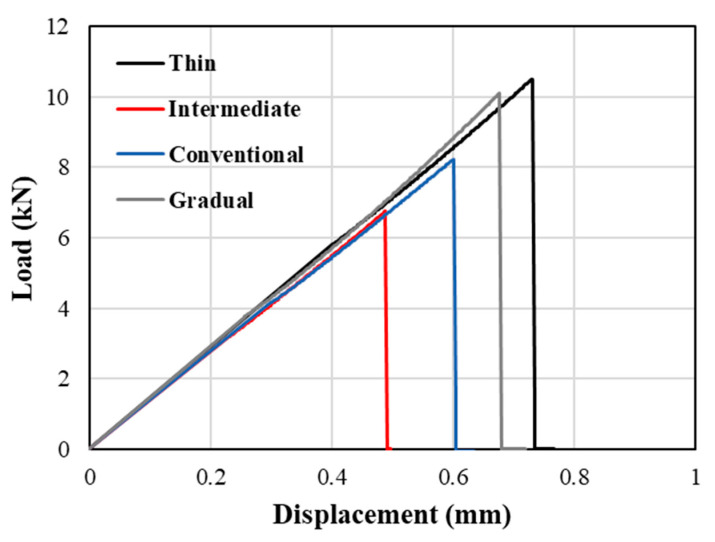
Representative experimentally obtained load–displacement curves.

**Figure 15:**
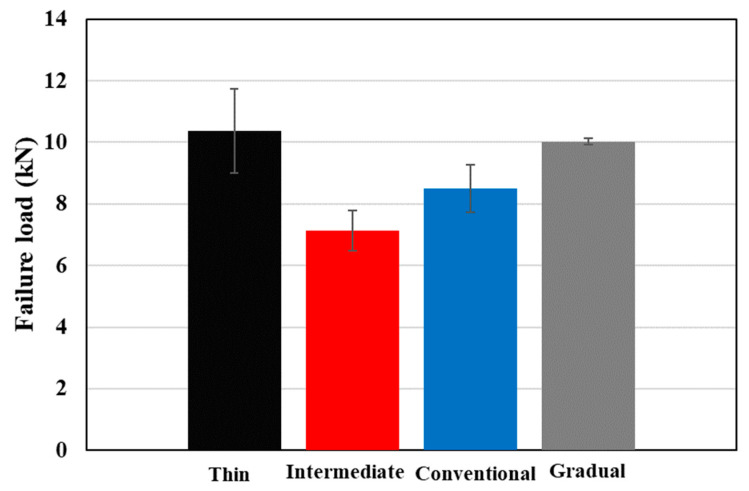
Effect of using thin plies gradually throughout the thickness of laminates on the transverse tensile strength.

Figure 14 displays experimentally obtained representative load–displacement curves from reference thin and intermediate thin ply, conventional, and gradual composite laminates. Notably, the gradual laminate displayed failure loads and corresponding displacement values comparable with thin-ply configurations, although these were higher than those observed in all other references. Generally, as illustrated in Figure 15, the gradual configuration demonstrated a 40% and 18% enhancement in failure load when compared to the reference intermediate thin ply, and conventional composite benchmark, respectively. While the thin thin-ply and gradual composite laminate exhibited comparable failure loads, it is important to note that the production of the gradual composite laminate is more cost-effective and less time-consuming than the manufacturing process for the reference thin thin-ply laminate. Despite the thin thin-ply reference laminate’s failure load being roughly higher than that of the gradual configuration, manufacturing a 3 mm thick laminate requires the layering of 200 plies. In contrast, the gradual configuration achieves a similar strength with only a quarter of the thin thin plies (50 plies). Furthermore, the standard deviation of results for the thin-ply configuration demonstrates comparatively higher values compared to other configurations, attributed to challenges in manufacturing and increased imperfections. This difficulty occurs because of the super-thin layers, making it challenging to manufacture using hand lay-up techniques. Hence, the proposed gradual configuration enables the utilization of thin thin plies to their fullest potential with reduced effort and material usage.

Building upon the prior investigations conducted by this research group and drawing from established knowledge in the field [6], it was observed that thin ply exhibits more ductile behaviour when compared to the conventional composite. Consequently, the enhancement in failure load under transverse tensile loading is primarily attributed to an increase in the laminate’s ductility due to the inclusion of thin ply in the hybrid configuration. Moreover, the gradual alteration of layer thickness throughout the laminate’s thickness leads to a more consistent and gradual transfer of load. Consequently, this leads to greater strength in the gradual laminate compared to both the reference laminate and the previously studied configuration that used thin plies as reinforcement locally, as examined by the authors [6].

**Figure 16:**
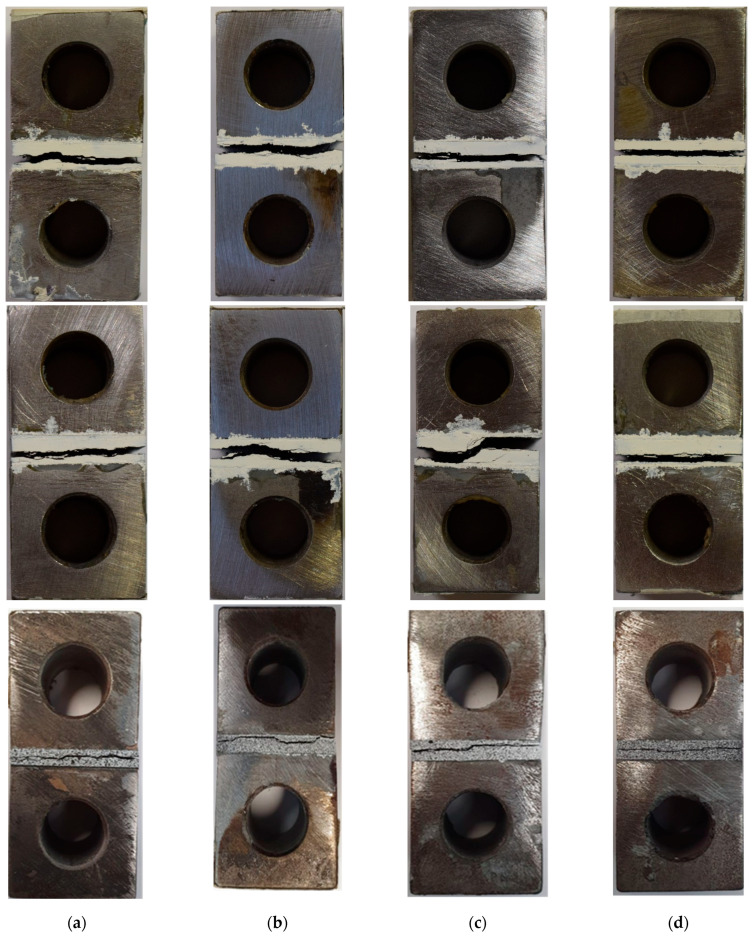
Damage mechanism for (**a**) thin, (**b**) intermediate, (**c**) conventional, and (**d**) gradual configurations.

The examination of crack paths and failure mechanisms in each of the configurations depicted in Figure 16 led to the observation that the failure initiation mechanisms were quite consistent across all configurations. For the reference configurations, the crack had the potential to extend throughout the thickness and reach the interface between the steel block and the composite laminate. In contrast, in most cases, crack initiation primarily occurred at the laminate’s corners, subsequently propagating towards the interface between the thin plies and the conventional composite in the gradual laminates. The presence of this barrier, created by the thin plies, is suggested as a contributing factor to the higher strength of the gradual laminate. Consequently, this barrier reduced the crack growth and provided resistance against crack propagation, which resulted in an increase in the failure load.

Additionally, scanning electron microscope (SEM) micrograph images also revealed a reduction in resin and fiber-rich regions within the thin ply laminates compared to the conventional ones [6,36]. Uniformly dispersed fibers and consequently altered resin distribution within the composite material could contribute to enhancing the obtained strength by decreasing the stress concentration in micro scales and also creating a more complex expected crack path throughout the thickness for an initiated crack.

### 4.2. Numerical Results and Discussion

**Figure 17:**
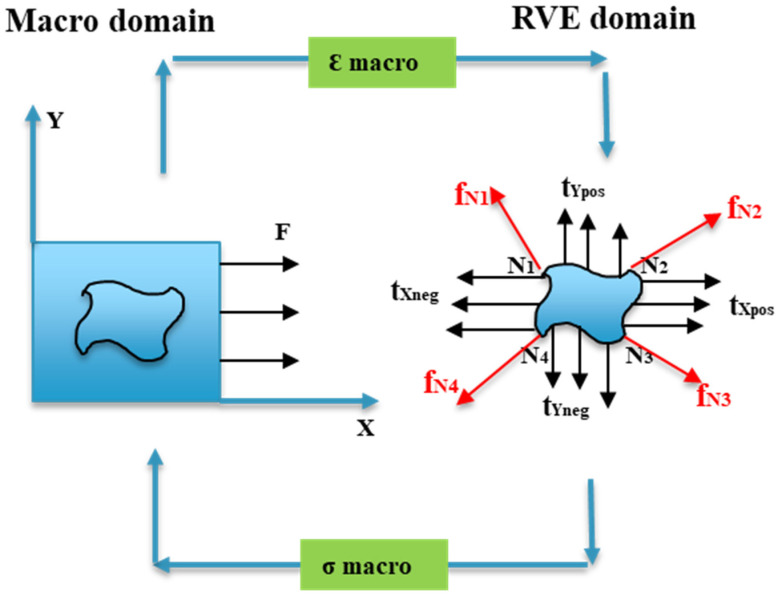
A 2D representation of macro-to-micro scale transitions for a heterogeneous material.

Based on Figure 17, the macro scale and RVE micro scale with dimensions named as L_Macro_ and L_RVE_ correspond to each scale. It is assumed that the order of L_Macro_ is significantly greater than L_RVE_ (L_RVE_/L_Macro_ << 1). Hence, to ensure the RVE can be representative of the entire domain in the macro scale, specific boundary conditions should be applied to the model. The load applied at the macro scale induces traction at each corner node and side of the RVE. Computational homogenization is employed to establish the connection between the macroscopic and microscopic fields, aiming to determine the strength in the macro scale domain [37,38]. In the periodically deformed domain of the RVE, denoted as the RVE domain in Figure 17, we can examine the stress state (*σ*) at a specific point, *x*, within the volume (*V*) enclosed by the RVE domain. This means we are analyzing the stress conditions at a particular location inside the volume defined by the periodically deformed RVE. It asserts that the outward flux of the stress field through a specified surface of the periodically deformed RVE domain is equivalent to the integral volume of the divergence of the region enclosed by that surface as presented in Equation (9) [26].

(9) $$\int_{V}\nabla .(\sigma ⊗x)dV=\int_{V}V[x⊗\nabla .\sigma +\nabla x^{T}\sigma ]dV=\int_{V}\sigma dV$$

Rewriting Equation (4) by incorporating the integral over the surface area results in the creation of Equation (10), as shown below:

(10) $$\int_{V}\sigma dV=\int_{A}(\sigma ⊗x).n_{s_{np}}dA=\int_{A}x⊗t_{s_{np}}dA$$

where *t_Snp_* = *σ_nSp_*. Finally, the volume-averaged stress within the periodically deformed RVE domain, as shown in Figure 17, becomes Equation (11).

(11) $$\langle \sigma \rangle =\frac{1}{V}\int_{V}\sigma dV=\frac{1}{V}\int_{A}x⊗t_{s_{np}}dA$$

A comparable formulation for volume-averaged stresses, denoted as <*σ*>, has been derived by Nemat-Nasser et al. [37]. The volume-averaged stresses within the RVE now become Equation (12).

(12) $$\langle \sigma \rangle =\frac{1}{V}[x_{1}⊗f_{N1}+x_{2}⊗f_{N2}+x_{3}⊗f_{N3}+x_{4}⊗f_{N4}]$$

In simpler terms, the last equation characterizes the volume-averaged stress within a periodically deformed RVE domain. This determination is made by considering the virtual work contributions originating from four retained nodal forces and the displacements of the 2D RVE domain. 

**Figure 18:**
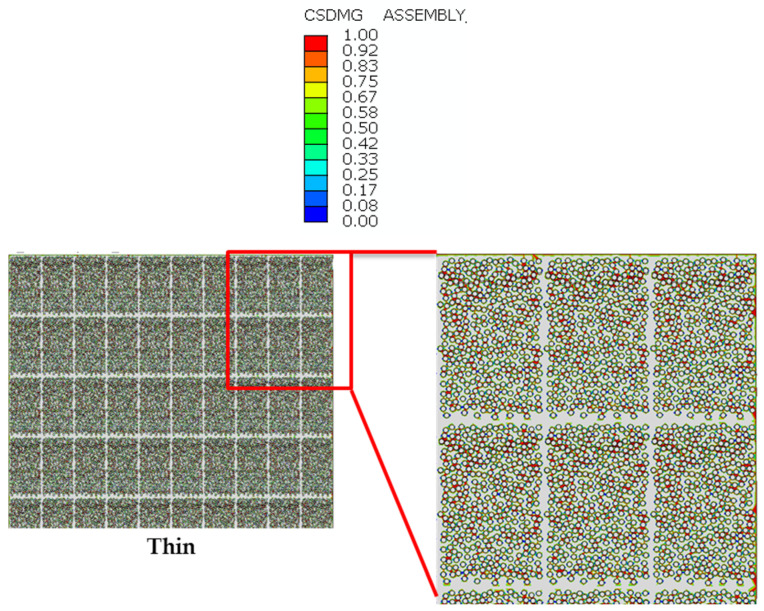

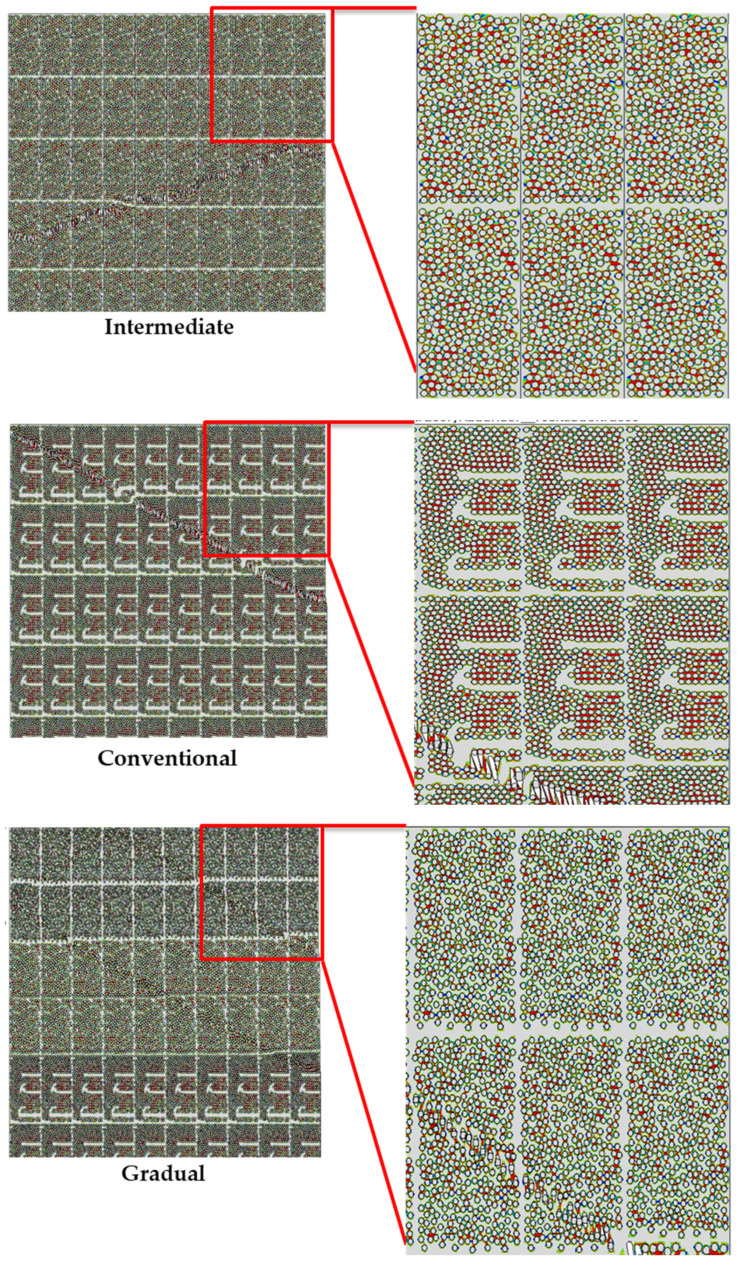
Fiber/matrix interface failure.

**Figure 19:**
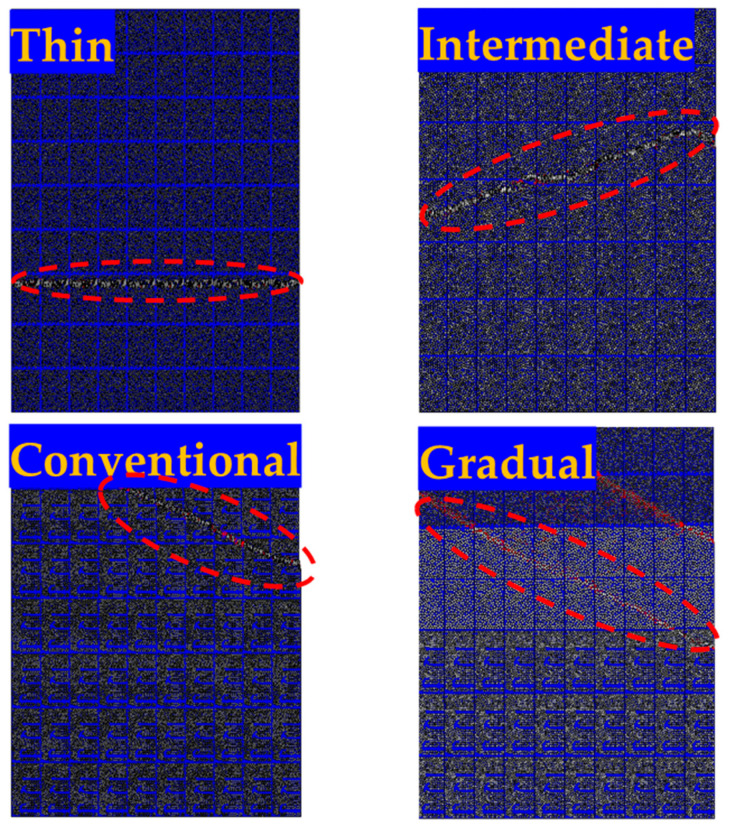
AC YIELD parameters for all configurations.

The fiber/matrix interface cohesive failure parameter and also the AC YIELD (actively yielding) provide a yes/no flag (1/0 on the output database), which indicates whether the material is currently yielding or not. The cohesive damage parameters (CSDMG) are illustrated in Figure 18. It is evident that for a given applied displacement, the percentage of interface cohesive damage is lower in gradual and thin configurations compared to other configurations. This visually confirms that gradual and thin thin-ply configurations may exhibit greater strength. Figure 19 displays the AC YIELDT values for all manufactured configurations, which can be considered as a parameter used to show the proportion of failure in all configurations. The dashed red line depicted in Figure 19 represents the failure corresponding to the AC YIELD parameter.

**Figure 20:**
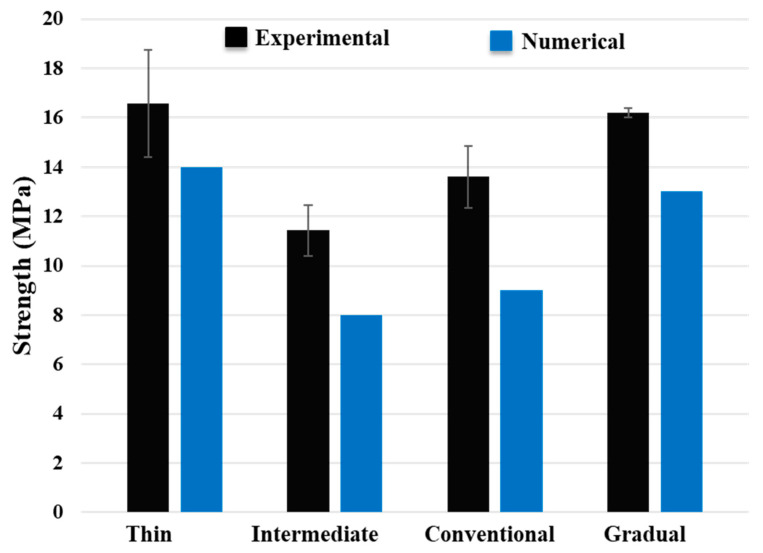
Comparison between numerical and experimentally obtained transverse tensile strength for different configurations.

The numerical results presented in Figure 20 demonstrate that the gradual configuration, incorporating 25% thin thin plies and 25% intermediate thin plies, exhibits a higher failure load compared to the conventional and intermediate thin-ply configurations. This improvement is attributed to enhanced ductility and well-distributed fibers, leading to a complex crack path. The discrepancy observed between the numerical and experimental results can be attributed to the RVE model and various parameters, such as applied boundary conditions, dimensions, and fiber distribution, all of which influence the behaviour of the RVE.

## 5. Conclusions

In this work, composite laminates that were gradually modified throughout the thickness with the use of thin plies were studied. Various combinations of thin plies and conventional composites were considered. Experimental assessment confirmed that gradually modifying composite laminates using thin plies enhances the transverse tensile strength compared to conventional composites. Although thin thin plies showed comparable strength with the gradual configuration, achieving the same thickness for the laminate requires only a quarter of the layers used in manufacturing the thin thin-ply configuration. This makes the gradual configuration cost-effective and reduces manufacturing defects. These findings highlight the potential benefits of gradually modifying composite laminates. A numerical 2D RVE model was created for each configuration in order to compare the strength of reference composite laminates with gradually modified configurations. The study employed concrete damage plasticity and cohesive zone models for accurate analysis. The numerical results indicated that the selected boundary conditions and algorithm for randomly creating fiber distribution can be effectively utilized in RVEs. Elastoplastic simulations also revealed that gradual modification resulted in a higher strength, indicating a positive impact on the composite’s strength characteristics.

## Figures and Tables

**Table 3:** Cohesive parameters (fiber/matrix interface) [28,31,32].

Tensile Strength (MPa)	Shear Strength (MPa)	*G_n_* (J/m^2^)	*G_s_* (J/m^2^)	$\eta_{BK}$
45	63	2	30	1.25

## Data Availability

Data are contained within the article.
